# Esophageal Trachea, a Unique Foregut Malformation Requiring Multistage Surgical Reconstruction: Case Report

**DOI:** 10.3389/fped.2020.605143

**Published:** 2020-11-20

**Authors:** Roberto Tambucci, Océane Wautelet, Astrid Haenecour, Geneviève François, Christophe Goubau, Isabelle Scheers, Marin Halut, Renaud Menten, Sandra Schmitz, Caroline de Toeuf, Thierry Pirotte, Beelke D'hondt, Raymond Reding, Alain Poncelet

**Affiliations:** ^1^Pediatric Surgery and Transplantation Unit, Department of Surgery, Saint-Luc University Clinics, Brussels, Belgium; ^2^Pediatric Intensive Care Unit, Emergency Department, Saint-Luc University Clinics, Brussels, Belgium; ^3^General Pediatric Unit, Department of Pediatrics, Saint-Luc University Clinics, Brussels, Belgium; ^4^Pediatric Pneumology Unit, Department of Pediatrics, Saint-Luc University Clinics, Brussels, Belgium; ^5^Pediatric Gastroenterology and Hepatology Unit, Department of Pediatrics, Saint-Luc University Clinics, Brussels, Belgium; ^6^Pediatric Radiology Unit, Department of Radiology, Saint-Luc University Clinics, Brussels, Belgium; ^7^Otolaryngology Unit, Department of Surgery, Saint-Luc University Clinics, Brussels, Belgium; ^8^Pediatric Anesthesiology Unit, Emergency Department, Saint-Luc University Clinics, Brussels, Belgium; ^9^Pediatric Cardiac and Thoracic Surgery Unit, Department of Surgery, Saint-Luc University Clinics, Brussels, Belgium

**Keywords:** foregut malformations, esophageal malformations, tracheal malformations, tracheomalacia, tracheoplasty, tracheal stenting, long-gap esophageal atresia

## Abstract

Abnormal connections between the esophagus and low respiratory tract can result from embryological defects in foregut development. Beyond well-known malformations, including tracheo-esophageal fistula and laryngo-tracheo-esophageal cleft, rarer anomalies have also been reported, including communicating bronchopulmonary foregut malformations and tracheal atresia. Herein, we describe a case of what we have called “esophageal trachea,” which, to our knowledge, has yet to be reported. A full-term neonate was born in our institution presenting with a foregut malformation involving both the middle esophagus and the distal trachea, which were found to be longitudinally merged into a common segment, 3 cm in length, located just above the carina and consisted of esophageal tissue without cartilaginous rings. At birth, the esophagus and trachea were surgically separated via right thoracotomy, the common segment kept on the tracheal side only, creating a residual long-gap esophageal atresia. The resulting severe tracheomalacia was treated via simultaneous posterior splinting of such diseased segment using an autologous pericardium patch, as well as by anterior aortopexy. Terminal esophagostomy and gastrostomy were created at that stage due to the long distance between esophageal segments. Between ages 18 and 24 months, the patient underwent native esophageal reconstruction using a multistage traction-and-growth surgical strategy that combined Kimura extra-thoracic esophageal elongations at the upper esophagus and Foker external traction at the distal esophagus. Ten months after esophageal reconstruction, prolonged, refractory, and severe tracheomalacia was further treated via anterior external stenting using a semitubular ringed Gore-Tex® prosthesis, through simultaneous median sternotomy and tracheoscopy. Currently, 2 years after the last surgery, respiratory stabilization, and full oral feeding were stably achieved. Multidisciplinary management was crucial for assuring lifesaving procedures, correctly assessing anatomy, and planning for multiple sequential surgical approaches that aimed to restore long-term respiratory and digestive functions.

## Introduction

Anomalies in embryologic foregut development can result in malformations of the upper digestive segments and respiratory tracts. A wide spectrum of abnormal connections between the esophagus and trachea or bronchi has been described, ranging from well-known malformations, including tracheoesophageal fistula and laryngo-tracheo-esophageal cleft, with or without esophageal atresia, to extremely rare and more complex malformations, such as communicating bronchopulmonary foregut malformations (CBPFMs) and tracheal atresia (1, 2). In 1976, Dietrich Kluth published a classification system of 10 types and several sub-types of foregut malformations, in which he drew the 96 different forms described up until that point (3). Subsequently, several case reports and small series regarding foregut malformations have been published (4).

Herein, we report the medical history of a full-term female neonate born with a foregut malformation characterized by the presence of a 3-cm common segment involving the middle esophagus and distal trachea that was located just above the carina and consisted of esophageal tissue without cartilaginous rings. This unique case, which, to our knowledge, has yet to be described, required challenging diagnostic, medical, and surgical management in order to assure lifesaving procedures, properly assess anatomy, and achieve long-term respiratory and digestive functions.

## Case Description

**Figure 1:**
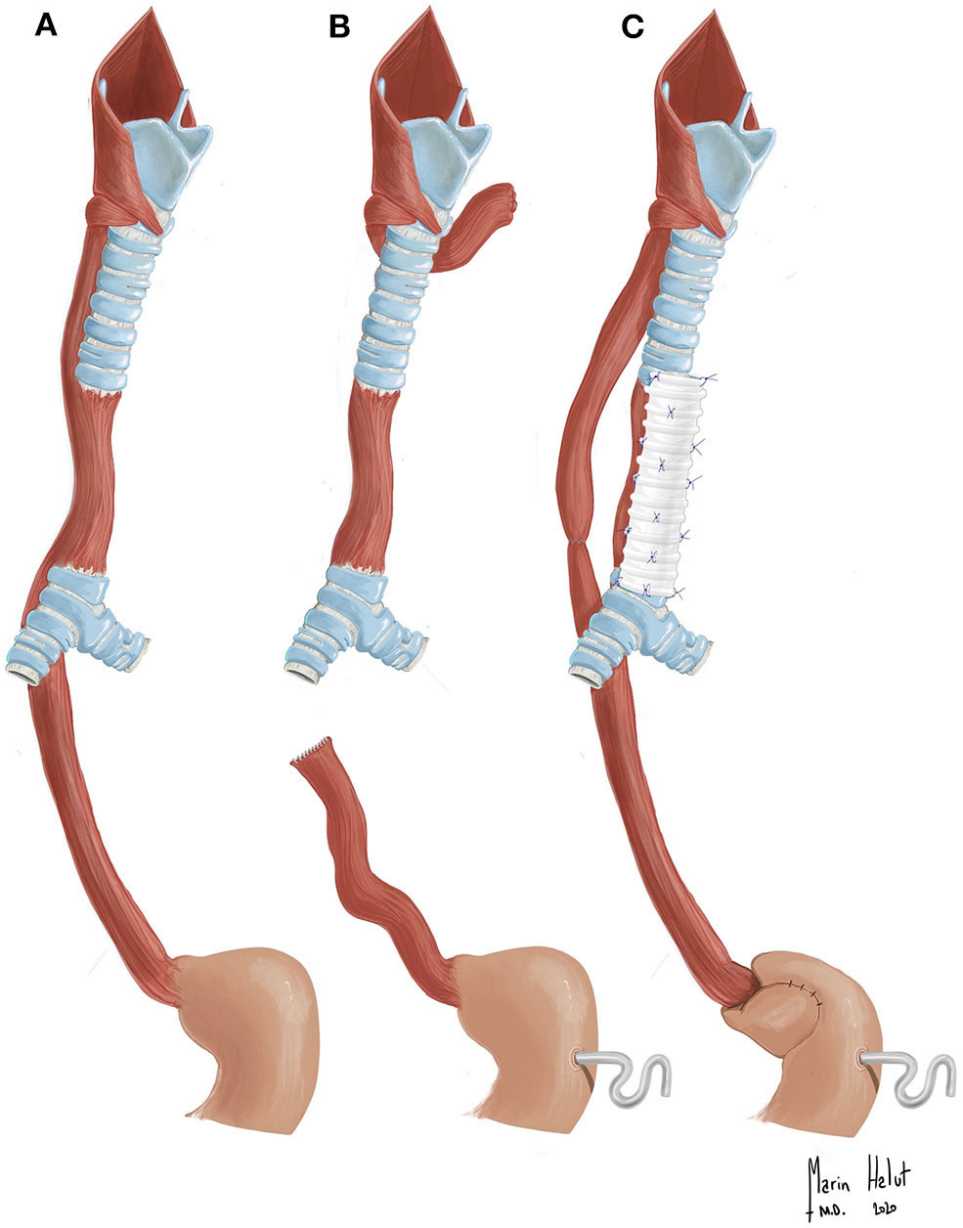
**(A)** Drawing of the patient's native malformation, which is characterized by the common segment involving both the middle esophagus and distal trachea. **(B)** Drawing after the first surgery. The common segment was kept on tracheal side. The esophagus disconnected right above and below this segment. Left cervical esophagostomy, Stamm gastrostomy, posterior tracheal splinting, and anterior aortopexy were performed (not depicted). **(C)** Drawing after multistage esophageal and tracheal reconstruction. Native esophageal reconstruction (end-to-end anastomosis) after performing the traction-and-growth strategy, Nissen fundoplication, and; anterior tracheal external splinting using a Gore-tex® stent were performed.

A female neonate was born from non-consanguineous healthy parents at Saint-Luc University Clinics, Brussels, Belgium, with a gestational age of 38 weeks and a birth weight of 2,770 g. A prenatal ultrasound detected right kidney agenesis and suspected aortic coarctation. At birth, VATER association was identified based on the combination of kidney agenesis, anorectal, and tracheo-esophageal malformations. Cardiac anomalies were not confirmed on neonatal echocardiography. Immediately after birth, the patient developed respiratory distress requiring tracheal intubation and mechanical ventilation. Attempts to place a nasogastric tube, which was first introduced in the left bronchus, according to chest X-ray, and eventually placed in the stomach, revealed the absence of esophageal atresia and, at first, suggested the presence of a laryngo-tracheo-esophageal cleft. Combined esophageal and laryngotracheal endoscopies at day 2 of life showed normal laryngo-pharyngeal anatomy, but revealed a large communication between the esophagus and distal trachea located just above the carina, where both nasogastric and tracheal tubes were simultaneously visible. At day 5 of life, the patient underwent right lateral thoracotomy through the 4th intercostal space. During surgery, middle esophagus and distal trachea were found to be merged in a common segment that measured 3 cm in length and was located just above a normal and solid carina. Based on the absence of cartilaginous rings, the segment consisted of esophageal tissue (Figure 1A). The cartilage gap was deemed too long for primary tracheal reconstruction. Therefore, we decided to keep the common segment on the tracheal side only, primarily to preserve respiratory continuity. For this purpose, the esophagus was disconnected right above and below this segment, and the superior and inferior stumps were closed on the tracheal side to prevent air leaks (Figure 1B). A longitudinal septum was not found inside the common segment. Due to the absence of cartilaginous rings, with the aim to prevent airway collapse, posterior splinting using a glutaraldehyde-fixed autologous pericardial patch and anterior aortopexy, consisting in three reinforced stitches placed on the brachiocephalic trunk/ascending aorta and attached to the periosteum of the posterior sternal table, were simultaneously performed through the same thoracotomy incision. Residual esophageal segments were too far apart from each other (about 4 cm) to attempt primary esophageal reconstruction. Consequently, the creation of left cervical terminal esophagostomy, the closure of the distal esophageal segment, and the creation of a Stamm feeding gastrostomy were performed, which created a secondary long-gap esophageal atresia (Figure 1B).

**Figure 2:**
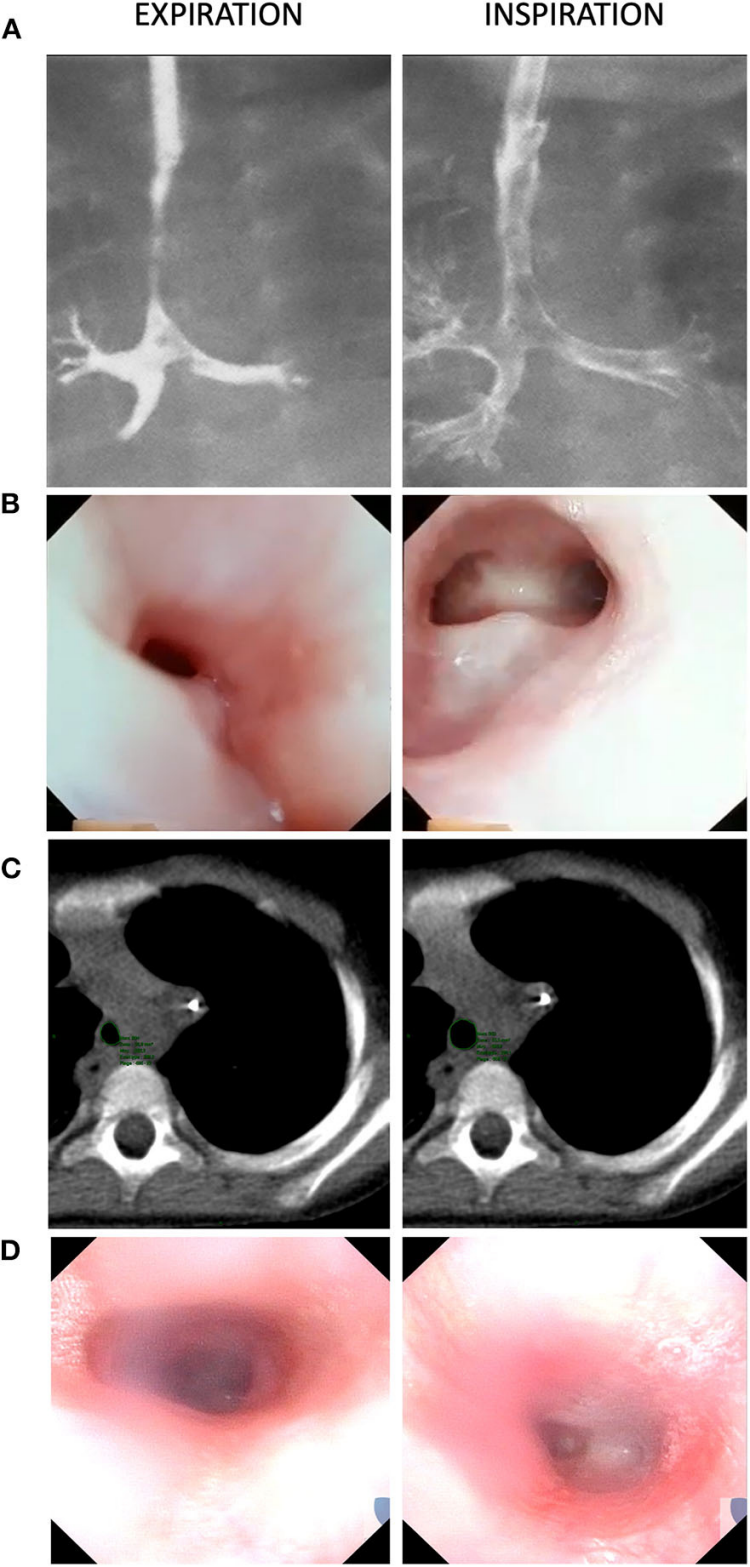
Expiratory airway collapse of the neo-tracheal segment, as observed via: **(A)** Tracheobronchography (Day 40). **(B)** Tracheoscopy (Day 865). **(C)** Dynamic computed tomography (CT) scan (Day 870). **(D)** Tracheoscopy (Day 1,798). Both tracheobronchography and tracheoscopy (images **A,B**), comparing expiration and inspiration frames, allow to appreciate esophageal-like appearance of the unsupported segment (due to the absence of cartilaginous rings), as also confirmed at CT scan (image **C**). At tracheobrochography (image **A**—inspiration frame), it is possible to appreciate a sort of diverticulum, just above the unsupported segment, which represents the site of separation of the proximal esophageal portion from the common segment. Similarly, in tracheoscopy views (images **B,D**—inspiration frames), a fovea on the posterior neo-tracheal wall, just above the carina, results from the separation of the distal esophageal portion. The most recent tracheoscopy (image **D**) confirms the absence of tracheal collapse 2 years after external stenting surgery.

After surgery, the child experienced prolonged episodes of respiratory obstruction symptoms due to severe tracheomalacia. Mechanical ventilation was maintained for 82 days during an intensive care unit stay of almost 3 months. Several diagnostic tests, including laryngo-tracheal endoscopy, tracheobronchography, and three-dimensional dynamic computed tomography (CT) confirmed the 360° expiratory collapse of the distal trachea (Figure 2A). As the child grew up, respiratory difficulties progressively improved. She was able to be weaned from non-invasive respiratory support and was discharged from the hospital at the age of 5 months, and was re-hospitalized twice during the subsequent 4 months due to respiratory and urinary infections, with no need for mechanical ventilation.

At the age of 14 months, with the patient's respiratory status deemed stable, she underwent fluoroscopic esophageal gap assessment under general anesthesia. For this assessment, contrast fluid was firstly injected through the gastrostomy fistula, allowing to visualize stomach and distal esophagus. Then, similarly, and a Hegar bougie was inserted in the stomach and guided into the distal esophageal segment, helping to estimate elasticity of esophageal tissue. The following distances between the distal esophagus and cervical esophagostomy were estimated in accordance with previously published methods (5): 7 vertebral bodies after contrast fluid injection and 4.5 vertebral bodies after applying mild boost on the Hegar bougie. Despite this long gap, we decided to avoid esophageal replacement and, instead, planned for native esophageal reconstruction by means of a multistage traction-and-growth strategy that aimed to induce tissue proliferation and elongation (6, 7).

For this purpose, the child first underwent two Kimura-Dessanti extrathoracic elongations at the age of 18 and 20 months, which lengthened the upper esophageal segment by 5–6 cm (8, 9). Then, redo right thoracotomy was performed at the age of 24 months, and Foker external traction on the distal segment was realized, which eventually reduced the residual gap until tension-free delayed end-to-end anastomosis was achieved 6 days later (10). The child remained electively intubated under pharmacological muscular paralysis for 4 more days after esophageal reconstruction to reduce the risk of anastomotic leakage, which was excluded via barium esophagogram at 8 days post-esophageal reconstruction.

Several episodes of respiratory obstruction recurred after tracheal extubation, which were temporarily managed via non-invasive support (Bi-level Positive Airway Pressure and Continuous Positive Airway Pressure [CPAP] support) and short periods of mechanical ventilation. Over 10 months ensuing esophageal reconstruction, the patient needed to be reintubated four times for a total of 23 days. Repeated tracheoscopies excluded vocal cords paralysis and detected a 360° collapse of the distal trachea, demonstrating the recurrence of severe tracheomalacia (Figure 2B). Three-dimensional dynamic CT confirmed persisting distal tracheomalacia with no signs of external compression by the esophagus (Figure 2C). Respiratory symptoms seemed to be associated with oral and gastric feeding, and gastroesophageal reflux disease, which was confirmed via 24-h esophageal multichannel intraluminal impedance and pH monitoring, was suspected to play a role. Consequently, the patient underwent Nissen fundoplication at the age of 30 months to correct hiatal hernia and prevent reflux episodes. Unfortunately, this surgery did not result in the significant improvement of respiratory symptoms. Accordingly, multidisciplinary decision was taken to manage tracheomalacia surgically again. The absence of cartilaginous rings at the tracheal bifurcation, the length of the unsupported airway wall, and the previous mediastinal surgeries were key reasons for choosing an external tracheal splinting strategy over other technical alternatives.

**Figure 3:**
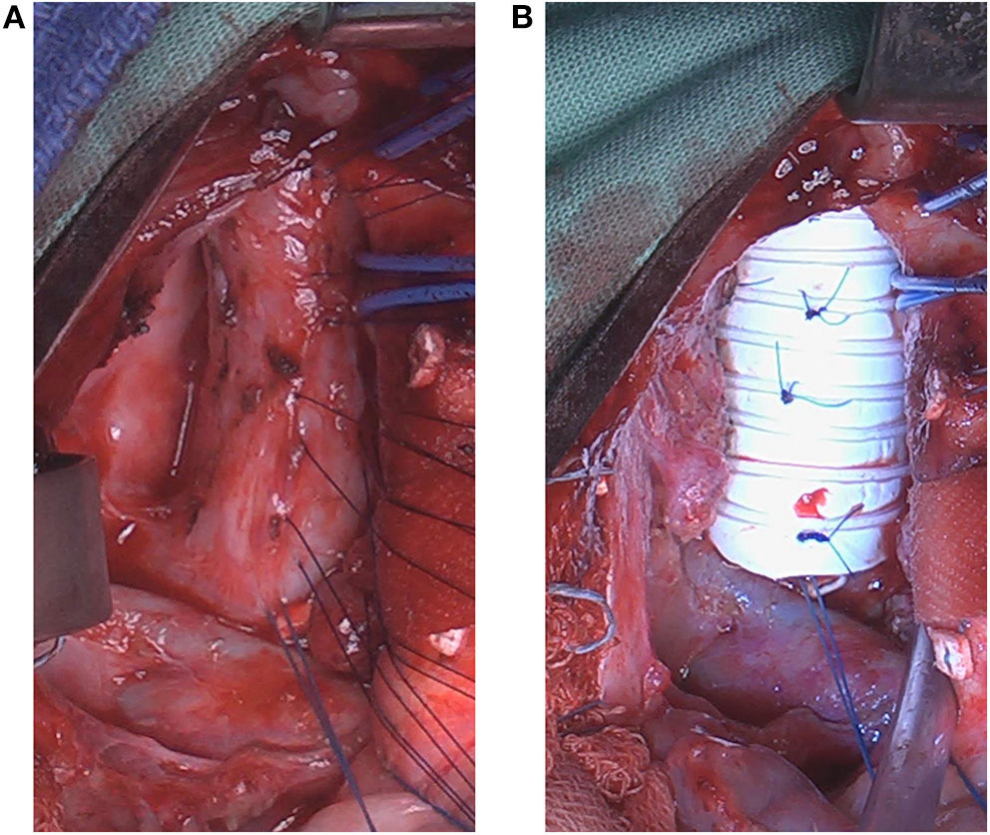
Intraoperative photos during anterior tracheal stenting/splinting (Day 1,012). **(A)** The anterior neo-tracheal wall is exposed through median sternotomy. Several rows of extra-mucosal 5/0 non-resorbable stitches were applied. **(B)** The stitches are anchored to the ringed hemi-circular Gore-tex® stent in order to suspend the anterior neo-tracheal wall and prevent airway collapse.

At 10 months after esophageal reconstruction, the patient underwent anterior sternotomy, with the purpose to anchor the anterior tracheal wall to an external semitubular rigid stent, using a circular ringed Gore-tex® prosthesis (diameter, 16 mm; length, 40 mm), which was longitudinally cut to be used as a hemi-cylinder. Several rows of extra-mucosal 5/0 non-resorbable stitches were applied between the diseased tracheal segment and this external stent (Figures 3, 1C). This surgical procedure was performed under tracheoscopy guidance to assure the complete patency of the malacic segment (11). Additional anterior tracheopexy was performed in order to stabilize the segment just cephalad to the stent, after removal of the previous aortopexy stitches, since no longer effective. Such tracheal portion was anchored with two 2/0 non-resorbable stitches to the periostium of the posterior sternal table, one on each side.

Two days after this last surgery, the child could be separated from invasive ventilation and CPAP support was applied, and then progressively reduced, but maintained without oxygen during sleep. Additionally, oral feeding was quickly reintroduced, well-tolerated, and progressively increased. The patient was discharged at 40 days after the last surgery. Full oral feeding was achieved at about 9 months post-surgery.

Currently, at 5 years of age, the child has exhibited normal neurodevelopment and growth (z-score: −0.6 SD for weight, +0.4 SD for height), eaten exclusively by mouth, only undergoes CPAP support during the night (+7 cmH2O), and is enjoying school every day with no restriction on normal physical activities. Endoscopic follow-ups showed no recurrence of tracheomalacia (Figure 2D). Further, she has not developed any clinical, radiological, or endoscopic signs of esophageal anastomotic stricture. Gastrostomy fistula was closed surgically at the age of 5, after more than 1 year without any use. After tracheal surgery, she has crossed two winters and was hospitalized due to respiratory symptoms seven times, including just once in the last season, for a total of 36 days, temporarily needing CPAP support increase. However, mechanical ventilation was never applied during those occasions. Chronological medical history is summarized in Table 1.

**Table 1:** A summary of the patient's chronological medical history.

**Age**	**Event**	**Details**
Day 1	Respiratory distress	Tracheal intubation and mechanical ventilation
Day 2	Laryngotracheal and esophageal endoscopies	No laryngeal cleft observed Detection of large distal tracheo-esophageal communication
Day 5	First surgeries	Right lateral thoracotomy Common segment kept on the tracheal side Proximal and distal esophageal segments disconnected Distal esophageal segment closed Left terminal esophagostomy Stamm gastrostomy Posterior tracheal splinting Anterior aortopexy
Day 85	Discharged from ICU	With non-invasive respiratory support
Day 151	Discharged from hospital	Without any respiratory support
Day 438	Esophageal gap assessment	Gap length: 4.5 vertebral bodies (with boost) Length of hospital stay: 2 days
Day 531	1st Kimura operation	Proximal esophagus lengthened by 3 cm Residual gap length: 3 vertebral bodies (with boost) Length of hospital stay: 5 days
Day 608	2nd Kimura operation	Proximal esophagus lengthened by 2.5 cm Residual gap length: 1.5 vertebral bodies (with boost) Length of hospital stay: 4 days
Day 718	Foker operation	Redo right thoracotomy External traction on distal esophageal segment for 6 days
Day 724	Esophageal reconstruction	Redo right thoracotomy Tension-free delayed end-to-end esophageal anastomosis
Day 926	Nissen fundoplication	Laparoscopy
Day 1,012	External tracheal stenting and anterior tracheopexy	Sternotomy Intraoperative tracheoscopic view
Day 1,052	Discharge from hospital	CPAP during night with O2 Oral and gastric feeding Length of hospital stay (since esophageal reconstruction): 334 days
Day 1,798	Endoscopic assessments Surgical closure of gastrostomy	No tracheomalacia relapse No esophageal stricture Length of hospital stay: 4 days
Day 1,852	Most current follow-up	Respiratory stability (CPAP continued during night) Full oral feeding

*CPAP, Continuous positive airway pressure; ICU, intensive care unit; O_2_, oxygen*.

## Discussion

The lower respiratory tract, which includes the area between the larynx and lungs, embryologically originates from the foregut, firstly through the formation of the respiratory bud, and then by the action of the tracheo-esophageal septum (12–14). Consequently, the alteration of this embryological process can result in communicating malformations that might involve not only the esophagus and trachea, but also the pharynx, stomach, and biliary system, with the larynx, bronchi, and lungs (15–19).

In 1976, Kluth published the extensive “Atlas of Esophageal Atresia,” which remains a leading reference in the field. He described 96 types of foregut malformations, which were classified with 10 groups, including the large spectrum of esophageal atresia, with or without tracheo-esophageal fistula, as well as laryngo-tracheo-esophageal clefts, CBPFMs, and tracheal atresia (3). The malformation detected in our case was not recognizable among any of those described in the atlas and, to the best of our knowledge, has not been presented in further published case reports and small series (1, 4, 14–20). Congenital absence of tracheal or bronchial rings, sometimes associated with esophageal atresia and trifurcated carina, has been described in very few cases, but none of them presented a segmental fusion between esophagus and distal trachea (21, 22). In our case, initial early endoscopy and thoracotomy revealed the peculiar anatomy of a malformation characterized by the presence of a common tracheo-esophageal segment without cartilaginous rings (Figure 1A). As such, we called it an “esophageal trachea,” and we propose that this malformation might be considered a variant type of CBPFMs. Interestingly, although a carinal trifurcation has not been formally found at endoscopy (Figure 2B), the right upper lobe bronchus arises at the origin of the right main bronchus, just below the carina (Figure 2A).

From a surgical strategy perspective, at birth the main decision was to avoid splitting such common segment in two separate conduits, as for repair techniques in type IIIb and IV tracheo-esophageal clefts (23). We preferred to keep the common segment on the tracheal side only, thereby temporarily sacrificing esophageal continuity. At that stage, the following two main problems remained: the resulting tracheomalacia and secondary long-gap esophageal atresia.

Severe tracheomalacia resulted from the esophageal origin of a 3-cm segment of the reconstructed trachea, cartilaginous rings being absent (Figure 2). The initial approach for preventing collapse included posterior splinting and anterior aortopexy, which were performed after a few days of life, simultaneously with esophageal separation. Then, at the age of 3 years, external stenting and anterior tracheopexy were also performed when tracheomalacia relapsed after esophageal reconstruction. This strategy was chosen over other surgical options, including tracheostomy, slide tracheoplasty, posterior tracheopexy, and internal stenting, which have been estimated to be potentially ineffective, unfeasible, or associated with high risks of morbidity for our particular case. Tracheostomy, associated with long-term ventilation, has been widely used in the past for severe cases of tracheomalacia. Currently, it is not recommended as primary approach, due to well-known related complications, particularly in young children, including the risk of secondary tracheomalacia (24). In our case, tracheostomy was excluded even due to the risk of incomplete effectiveness, since the malacic segment was limited to distal trachea. Segmental tracheal resection with slide tracheoplasty has been proposed for removal of short segment of tracheomalacia (21, 22, 25, 26). In our case, this approach was considered, but eventually estimated to be not feasible due to the length of the malacic segment, as well as because of the short residual distance from the carina. Anterior and/or posterior tracheopexy have been recently proposed and quickly gained wide popularity (27–29). Posterior pexy is realized by mobilizing the esophagus laterally, allowing to suture the posterior tracheal wall to the anterior longitudinal ligament of the spine. This technique was not considerable in our case due to the original characteristics of the malformation, namely the fact that the malacic segment, being of esophageal origin, was already close to the column, with esophagus being reconstructed laterally on the right side. Internal stenting, using metallic, silicon or bioresorbable materials, is an attractive strategy, particularly because of low invasiveness. It was firstly proposed for adult patients (30), but it never reached appreciation for the use in pediatric cases, due to the higher rate of complications, such as migration and erosion, as well as because the small size of pediatric airways and the need for growth (31–34). External stenting can be performed using either autologous or prosthetic materials. The use of autologous rib cartilage graft was reported for long segments of severe tracheomalacia in children, aiming to stabilize the trachea, by fixing it to cartilaginous rings (35, 36). Several prosthetic materials, even bioresorbable, have been proposed with the same purpose (37). For our case, we decided to use ringed polytetrafluoroethylene, as described by Ando et al. in 2016, who treated 98 children with good long-term results, with their first case being performed in 1997 (11). They reported using two separate pieces of polytetrafluoroethylene, one for the anterior wall and one for the posterior membrane, which were not sutured together, thereby allowing for tracheal growth over time. In our case, we only needed to act on the anterior wall, so a single semitubular piece was used. Despite favorable long-term follow-ups having been previously reported, the potential risks of permanent foreign materials being implanted in children, including infections, erosion, and tracheal compression, remain a potential concern (38). In our case, after 26-month follow-up, the child progressively improved in terms of clinical and respiratory status, but instrumental follow-ups via endoscopies, CT, and functional tests are planned for her.

Additionally, we evaluated several options for delayed esophageal reconstruction. Initially, we considered colonic or gastric replacement, as estimating the length residual gap was too long for native esophageal preservation. However, concerns were expressed about the risks of placing a large graft, either the stomach or colon, in the posterior mediastinum, due to the possible compression on the malacic trachea. Moreover, a retrosternal route was not considered due to previous aortopexy. Therefore, native esophageal reconstruction was reconsidered as our first option, and a traction-and-growth strategy was planned to reduce the residual gap, inducing progressive elongation and proliferation of esophageal tissue (6, 7). Despite the Kimura and Foker techniques having been proposed several years ago (8, 10), their use has only recently gained popularity in the field, and the combination of both techniques has been reported in very few cases (39, 40). In this peculiar situation, we decided to reduce the gap as much as possible and aimed for an almost tension-free anastomosis, in order to avoid esophageal compression on the trachea. Consequently, the esophagus was reconstructed laterally on the right side, as described for posterior tracheopexy (28). At the time of definitive esophageal anastomosis, since the esophagostomy was created on the left side, crossing the cervical esophagus behind the trachea was necessary for reaching distal segment in the thoracic cavity. To avoid this maneuver, we recommend to rather create cervical esophagostomy in the right side. Moreover, we suggest that esophagostomy is fashioned under maximal tension, in order to preserve its length as much as possible, possibly creating a subcutaneous channel, when necessary; proximal esophagus might be protected by a synthetic patch around, aiming to help secondary dissection (9). Similarly, distal esophagus might be anchored to the anterior longitudinal ligament of the spine, in order to avoid retraction and progressive loss of tissue. Furthermore, thanks to those expedients, we believe that native esophageal reconstruction should be always considered as a first choice, even when previous surgeries had already been realized and the gap seems too wide (5, 41).

Concluding, before operating on any foregut malformation, including esophageal atresia, preoperative laryngo-tracheo-bronchoscopy should be considered for the early exclusion of rare anatomical conditions requiring specific management (42). The unique case we describe illustrates the need for precise anatomical assessments and the importance of the prolonged multidisciplinary management of complex diseases. Furthermore, tertiary-center referral is paramount for such peculiar patients.

## Data Availability Statement

The raw data supporting the conclusions of this article will be made available by the authors, without undue reservation.

## Ethics Statement

Written informed consent was obtained from the minor(s)' legal guardian/next of kin for the publication of any potentially identifiable images or data included in this article.

## Author Contributions

RT conceived the study and prepared the first draft of the manuscript. OW contributed to medical record review, literature revision, and preparation of first draft. RR and AP contributed to writing the first draft and to interpretation of data. AH, CG, and IS contributed to conception of the study and interpretation of data. GF, TP, and BD'h contributed to interpretation of data. RM contributed to selection of radiological images and to interpretation of data. MH drew the illustrations and contributed to selection of radiologic images. SS and CT contributed to selection on endoscopic images and to interpretation of data. All authors contributed to critically revising the manuscript, approved the final version of the manuscript, and the authorship list and take full responsibility for the manuscript.

## Conflict of Interest

The authors declare that the research was conducted in the absence of any commercial or financial relationships that could be construed as a potential conflict of interest.
